# Targeting Inflammation and Downstream Protein Metabolism in Sarcopenia: A Brief Up-Dated Description of Concurrent Exercise and Leucine-Based Multimodal Intervention

**DOI:** 10.3389/fphys.2017.00434

**Published:** 2017-06-22

**Authors:** Zhi Xia, Jason Cholewa, Yan Zhao, Hua-Yu Shang, Yue-Qin Yang, Kassiana Araújo Pessôa, Quan-Sheng Su, Fernanda Lima-Soares, Nelo Eidy Zanchi

**Affiliations:** ^1^Exercise Physiology and Biochemistry Laboratory, College of Physical Education, Jinggangshan UniversityJi'an, China; ^2^Department of Sports Medicine, Chengdu Sport UniversityChengdu, China; ^3^Department of Kinesiology, Coastal Carolina UniversityConway, SC, United States; ^4^Exercise Intervention and Health Promotion Hubei Province Synergy Innovation Center, Wuhan Sports UniversityWuhan, China; ^5^Department of Physical Education, Federal University of MaranhãoSão Luís, Brazil; ^6^Laboratory of Cellular and Molecular Biology of Skeletal Muscle (LABCEMME)São Luís, Brazil

**Keywords:** concurrent exercise, leucine, inflammation, protein metabolism, sarcopenia

## Abstract

Sarcopenia is defined as the progressive loss of muscle mass with age, and poses a serious threat to the physiological and psychological health of the elderly population with consequential economic and social burdens. Chronic low-grade inflammation plays a central role in the development of sarcopenia such that it alters cellular protein metabolism to favor proteolysis over synthesis, and thereby accelerates muscular atrophy. The purpose of this review is to highlight how exercise and nutrition intervention strategies can attenuate or treat sarcopenia. Resistance exercise increases not only muscle mass but also muscle strength, while aerobic exercise is able to ameliorate the age-related metabolic disorders. Concurrent exercise training integrates the advantages of both aerobic and resistance exercise, and may exert a significant synergistic effect in the aging organism. Higher protein intakes rich in the amino acid leucine appear to restore skeletal muscle protein metabolism balance by rescuing protein synthesis in older adults. There is good reason to believe that a multimodal treatment, a combination of exercise and increased leucine consumption in the diet, can combat some of the muscle loss associated with aging. Future research is needed to consolidate these findings to humans, and to further clarify to what extent and by which mechanisms protein metabolism might be directly involved in sarcopenia pathogenesis and the multimodal treatment responses.

## Introduction

Sarcopenia is a hallmark of the aging process. With the global increase in the elderly population, in part due to longer life expectancies and a higher prevalence of sarcopenia later in life, current estimates suggest that about 200 million people worldwide will be affected by year 2050 (Janssen, 2011). Accordingly, sarcopenia results in a heavy public health burden. A reduction in sarcopenia of just 10% would save $1.1 billion in health care savings by reducing major surgeries and hospitalization in patients (Sousa et al., 2016). Therefore, developing cost-effective interventions to improve muscle mass and strength in sarcopenic patients is vital for healthy aging, and is important to improve survival rates in the elderly and reduce social and economic burdens.

## Inflammation, protein metabolism, and potential targets

In healthy elderly subjects, the net loss of muscle mass seems to be the result of decreased rates of post-prandial muscle protein synthesis without alterations in the basal muscle protein synthesis (Volpi et al., 2001; D'Antona and Nisoli, 2010; Cholewa et al., 2017; Table 1), whereas in sarcopenic individuals (exhibiting a state of skeletal muscle atrophy), the progression toward sarcopenia ultimately involves alterations in skeletal muscle protein turnover, whereby rates of muscle protein breakdown (MPB) chronically exceed MPS (Trappe et al., 2004; Churchward-Venne et al., 2014). Presently, accurate methodologies have been utilized to measure protein synthesis *in vivo*, but when measuring protein degradation these methods have presented important limitations, especially when related to MPB (Zanchi et al., 2008). That is why we postulate that increased MPB is an important mechanism inducing sarcopenia although we recognize that the literature is still lacking in categorical measurements and comparisons of MPS and MPB simultaneously.

**Table 1:** Muscle protein synthesis in elderly and young subjects.

**Authors**	**Elderly**	**Young**
**1. Basal muscle protein synthesis (MPS) levels in healthy subjects**		
Chevalier et al., 2011	↔	↔
Cuthbertson et al., 2005	↔	↔
Dillon et al., 2011	↔	↔
Pennings et al., 2011	↔	↔
Rooyackers et al., 1996	↓^*^	↔
Toth et al., 2005	↓	↔
Volpi et al., 1998	↔	↔
**2. Post-prandial muscle protein synthesis (PP-MPS) levels**		
Balagopal et al., 1997	↑^*^	↑*↑↑*
Cuthbertson et al., 2005	↑^*^	↑*↑↑*
Guillet et al., 2004	↑^*^	↑*↑↑*
Katsanos et al., 2005	↑^*^	↑*↑↑*
Pennings et al., 2011	↑↑	↑↑
Short et al., 2004	↑^*^	↑*↑↑*
Volpi et al., 2000	↑^*^	↑*↑↑*

*1, Basal MPS levels in young and elderly subjects; 2, Post-prandial MPS levels (PP-MPS) in young and elderly subjects*.

* *Significant differences between elderly and young (P <0.05 or P <0.01)*.

The regulatory mechanisms explaining reduced MPS and increased MPB in sarcopenic skeletal muscle converge on the mammalian target of rapamycin (mTORC1), a protein kinase acting downstream of the PI3K-Akt pathway which is involved in both MPS and MPB regulation (Zanchi et al., 2008). With regard to protein degradation, tagging of proteins to be degraded in the proteasome molecular machine are performed by ubiquitin ligases which act downstream of the major protein degradation regulator FOXO (D'Antona and Nisoli, 2010). Although there are hundreds of ubiquitin ligases involved in MPB, two muscle specific E3 ubiquitin ligases, MuRF-1 and Atrogin-1, are required to induce both skeletal muscle atrophy and the inhibition of protein synthesis (Rom and Reznick, 2016).

Aging triggers a pro-inflammatory response that may be related to the gradual decline in physical activity and presents as low-grade chronic inflammation. The main source of pro-inflammatory molecules seems to be from adipose tissue (mainly the visceral adipose tissue), via increased activation and infiltration of immunological cells (usually macrophages and neutrophils). Activated macrophages may stimulate an increase in visceral adipose tissue lipolysis (also increasing circulating fatty free acids: FFA levels) via TNF-α/TLR-4 signaling. Whereas, increased FFA levels reinforce macrophages to increase the secretion of important pro-inflammatory molecules (such as TNF-α, IL-1, and IL-6), these molecules also affect the skeletal muscle, especially sedentary skeletal muscle. Indeed, increased TNF- α, IL-6, and FFA appears to contribute to skeletal muscle proteolysis activation and insulin resistance, two main factors involved in several types of muscle atrophy. The reason why increased MPB is the major determinant of muscle mass loss during sarcopenia seems to be linked to metabolic/endocrine alterations generated by co-morbidities which accompany sarcopenia. For example, co-morbidities (cancer, sepsis, heart failure) often lead to increased secretion of catabolic hormones such as cortisol, pro-inflammatory molecules such as cytokines, and increased ROS (reactive oxygen species) formation, which may result in increased muscle proteolysis (Crossland et al., 2008, 2010; Brocca et al., 2012; Santilli et al., 2015; Cholewa et al., 2017).

As discussed above, chronic systemic inflammation is an important intermediate in the process of sarcopenia, and can be regulated by pro-inflammatory mediators such as TNF-α, IL-1, and IL-6. Pro-inflammatory cytokines, especially the TNF-α and IL-6, regulate this process through a FOXO3a mediated pathway that activates the ubiquitin-proteasome system (Sente et al., 2016). These cytokines may work in a synergistic way to promote sarcopenia due to the cross-talk between inflammatory cells and organs, resulting in reduced protein synthesis and increased protein degradation, and ultimately leading to skeletal muscle loss and functional impairment. Thus, it can be seen that the increase in MPB and/or decrease in MPS directly leads to the age-related skeletal muscle loss, while chronic systemic inflammation accentuates the predisposition to sarcopenia (Budui et al., 2015).

## Combined exercise and leucine interventions for sarcopenia

The society for sarcopenia, cachexia, and wasting disease and some scholars previously released recommendations for the management of sarcopenia based on nutrition and exercise practice (Morley et al., 2010; Denison et al., 2015). The effectiveness of existing pharmaceutical and nutritional supplement interventions such as antioxidants and anti-inflammatory agents does not appear to either fully manage age related atrophy or has not been explored in detail (Reginster et al., 2016). Sarcopenia is a multifactorial syndrome, and given its multifaceted pathophysiology, a multimodal approach to sarcopenia management should be advocated. Over the past 30 years, the single intervention specifically aimed at increasing food intake, attenuating inflammation, or ameliorating protein balance disorders has provided limited results in the management of sarcopenia; research on the effectiveness of anti-inflammatory based single-mode treatment appears to be limited (Landi et al., 2013); high-dose hormone replacement therapy results are ambiguous and controversial because of the potential side-effects (Giannoulis et al., 2012); and no pharmaceutical drugs have been approved as potent treatments for sarcopenia (Watanabe and Miyagoe-Suzuki, 2015).

In recent years, the scientific community has progressively come to realize that etiologies containing inflammation, protein balance disorders, and physiologic “anorexia of aging” should be taken into account in the treatment of sarcopenia (Valerio et al., 2011). The main treatment objectives should include ensuring sufficient energy and protein intake, maintaining physical activity, and reducing chronic low-grade inflammation where present. To achieve this goal, the multimodal treatment includes exercise training, nutritional supplements, and pharmaceutical agents that may simultaneously counteract the aforementioned factors associated with atrophy and the adverse outcomes associated with sarcopenia (Dickinson et al., 2013; Denison et al., 2015).

The effects of exercise training on chronic low-grade systemic inflammation have been primarily studied via aerobic training. Aerobic exercise induces a reduction in visceral, subcutaneous, and local body fat volumes and has been shown to lower the production and accumulation of circulating pro-inflammatory cytokines and proteins (Park et al., 2008). Additionally, aerobic exercise can attenuate activation of the ubiquitin-proteasome pathway, inhibition of MPS via increased mTOR signaling, and may also promote skeletal muscle hypertrophy (Xia et al., 2016). On the other hand, resistance exercise, irrespective of nutrient type and feeding pattern, has been shown to significantly increase MPS, strength, and power in elderly men (Hasten et al., 2000). Resistance training also reduces systemic inflammation by inhibiting cytokines and inflammatory proteins in both aging and cachexic states, and decreases the activation of ubiquitin ligases and TLR-4 mRNA in trained muscle (Zanchi et al., 2009, 2010). Compared with aerobic or resistance exercise alone, performing both in the same exercise session or exercise training program (termed concurrent training) triggers more pronounced effects on promoting anti-inflammation, MPS, and functional improvements. Donges and colleagues found that only concurrent exercise resulted in ameliorated cytokine mRNA expression at 4 h post-exercise, and promoted similar acute myofibrillar MPS, protein signaling, and mRNA expression compared to resistance training alone (Donges et al., 2014). Stewart and colleagues showed that concurrent training increased muscle strength by 38% and resulted in significant reductions in CRP in both young and old physically inactive subjects when compared with pre-training values (Stewart et al., 2007), and Balducci's group demonstrated that regular participation in concurrent exercise exerts the greatest anti-inflammatory effects in comparison with aerobic exercise (low- and high-intensity conditions) by lowering systemic inflammatory cytokines, including IL-6 and TNF-α (Balducci et al., 2010). According to abovementioned results, there is good reason to believe that concurrent resistance and aerobic exercise training is an effective intervention to enhance aging skeletal muscle protein metabolism.

Although, sarcopenia occurs most severely in sedentary individuals, sarcopenia will also affect populations that participate in life-long physical activity. This suggests that regular exercise is necessary to avoid the occurrence and development of sarcopenia, but exercise alone may not be effective enough to completely prevent and/or delay sarcopenia. As previously discussed, healthy elderly subjects usually exhibit decreased post-prandial MPS (termed anabolic resistance) with no major signs of increased MPB (Dardevet et al., 2012; Cholewa et al., 2017). On the other hand, sarcopenic individuals usually show increased MPB rates (together with decreased MPS rates), dictating the majority of muscle atrophy that occurs (Cholewa et al., 2017). With these concepts in mind, it is becoming clear that treatment of sarcopenia must involve multi-modal strategies aiming neutralizing the low-grade inflammation but also promoting increased post-prandial MPS. In fact, as little as 2 weeks of inactivity has been shown to accentuate anabolic resistance in older adults (Breen et al., 2013), whereas the use of low-load resistance training has been shown to rescue the anabolic response to feeding during inactivity (Devries et al., 2015). We hypothesize that concurrent exercise will attenuate muscle atrophy to a greater degree via the aforementioned mechanisms; however, further research is needed to compare concurrent exercise to either aerobic or resistance training alone to promote decreased low-grade inflammation and muscle hypertrophy in sarcopenic individuals.

The essential amino acid leucine is one of the most studied potential nutritional interventions that may attenuate the development of sarcopenia (Valerio et al., 2011). Leucine promotes MPS via phosphorylation of the mTORC1, and its effects are more pronounced than other essential amino acids (Dickinson et al., 2013). Leucine has also been suggested to stimulate the appetite, decrease muscle proteolysis, and when combined with exercise, provides a synergistic protein synthetic response (Zanchi et al., 2008; Xia et al., 2016). Our group conducted an 8 week training study in pre-senescent mice. Mice in the experimental group received aerobic exercise training and 5% dietary leucine supplementation. Leucine supplementation in combination with exercise resulted in more significant effects on muscle mass, type II fiber cross-sectional area, and reductions in protein degradation compared to leucine or exercise alone (Xia et al., 2016). Moreover, we also found that the combined intervention inhibited an increase in inflammatory cytokines (TNF-α, IL-1β, and IL-6; Yang, 2014). These results suggests that leucine supplementation in combination with exercise training has the potential to promote protein synthesis, attenuate protein degradation, and inhibit inflammation.

**Figure 1:**
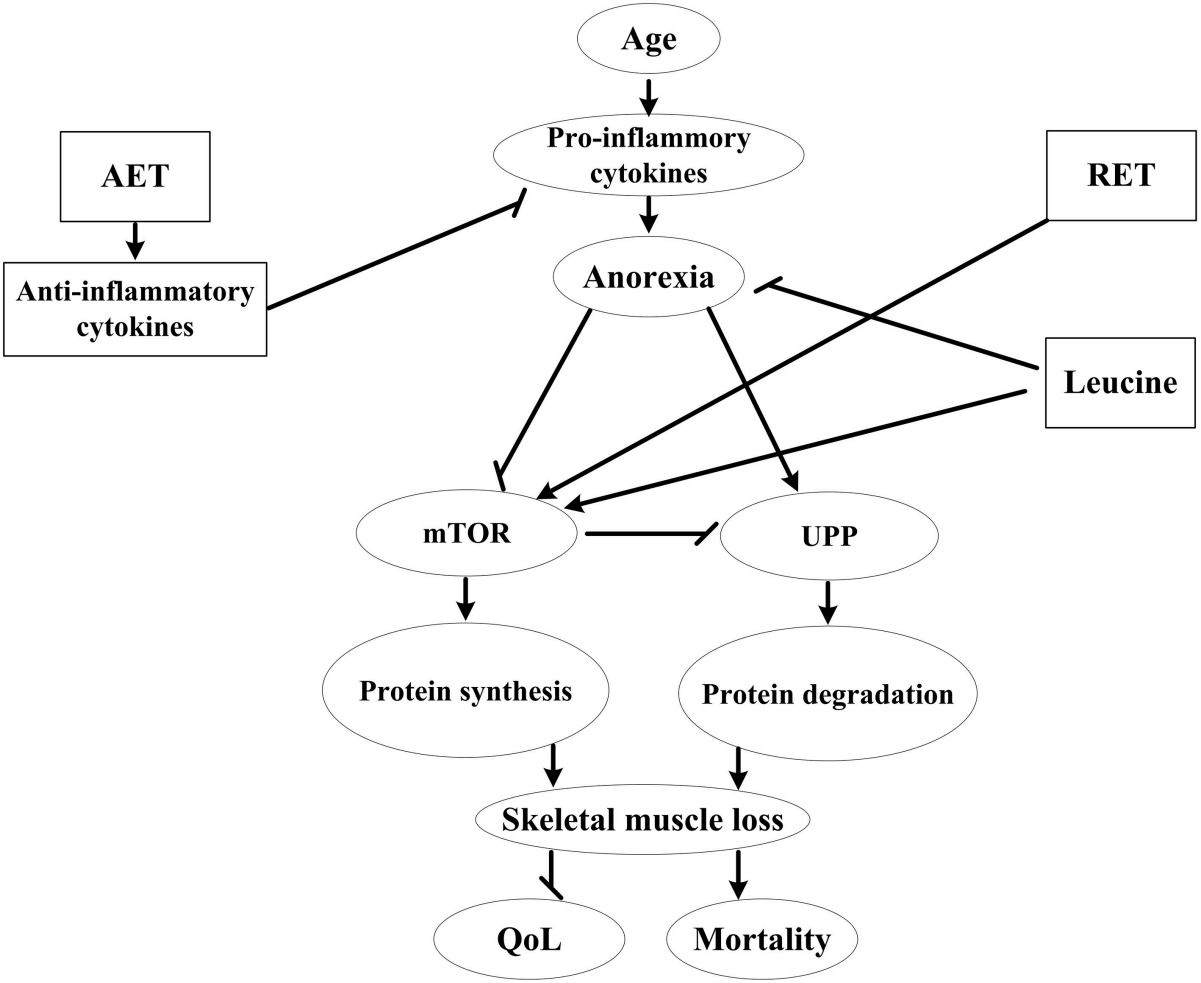
Effects of aerobic and resistance exercise training combined with leucine supplementation in sarcopenia. AET, aerobic exercise training; RET, resistance exercise training; mTOR, mammalian target of rapamycin; UPP, ubiquitin-proteasome pathway; QoL, quality of life; 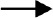 activate; 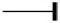 inhibit.

Currently, research on the effects of concurrent exercise training combined with leucine supplementation on sarcopenia is scarce. However, in light of aforementioned evidence and our previous results, we have reason to believe that by using the multimodal treatment, i.e., the combination of exercise and leucine supplementation, we can attenuate inflammation, suppress the secondary anorexia, reverse the negative protein balance, and potentially alleviate age-related skeletal muscle loss and improve impaired muscle function (Phillips, 2015). A schematic of how combined exercise and leucine supplementation may interact with inflammation and subsequent protein metabolism imbalance in the sarcopenic process is depicted in Figure 1.

**Figure 2:**
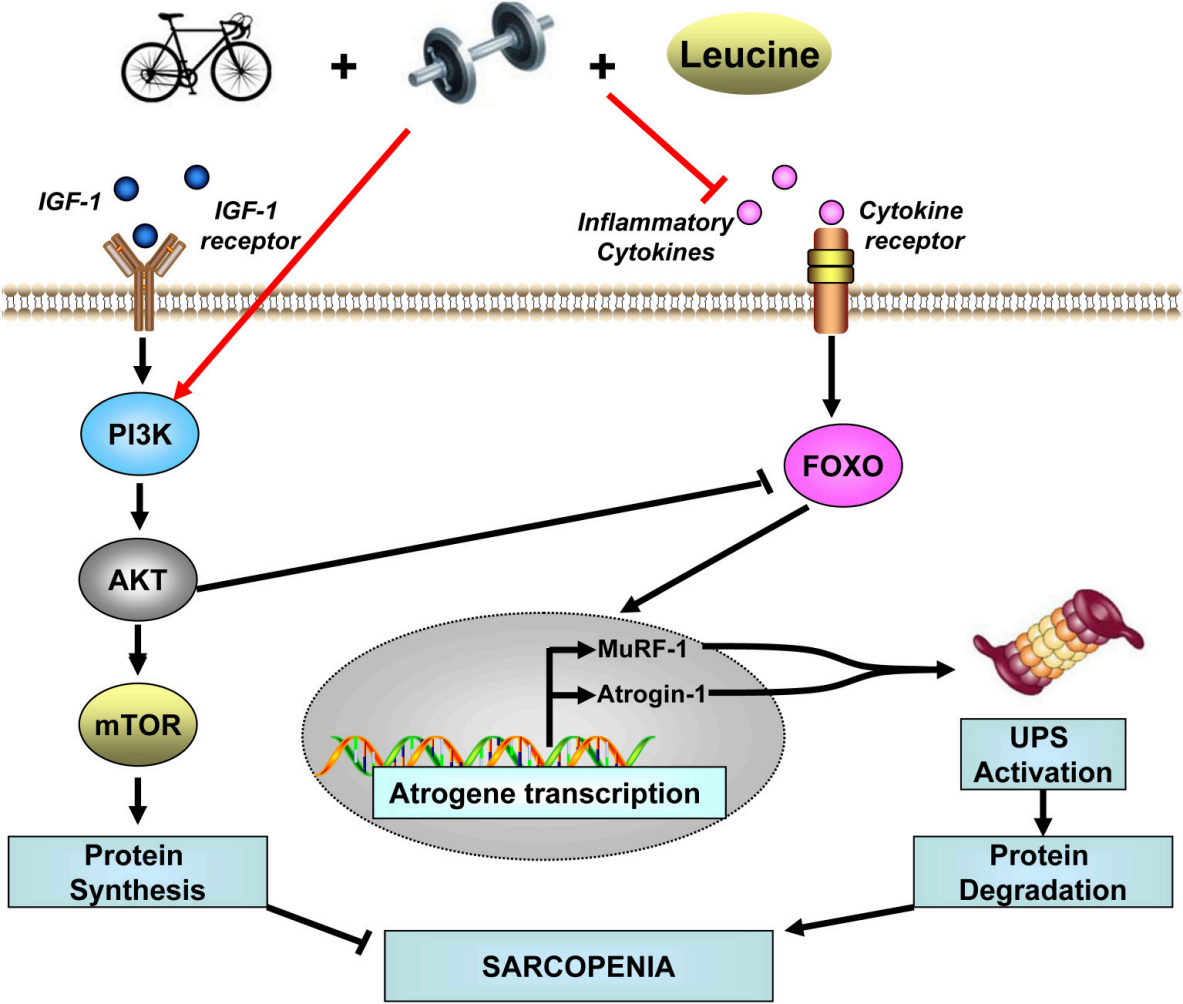
Possible effects of concurrent resistance and aerobic exercise and leucine supplementation to molecular mechanism that may accelerate MPS and decelerate MPB. IGF-1, Insulin-like growth factor 1; PI3K, phosphatidylinositol 3 kinase; Akt, serine/threonine protein kinase; mTOR, mammalian target of rapamycin; FOXO, Forkhead box protein O; MuRF-1, muscle ring finger-1; Atrogin-1, Muscle Atrophy F-box; UPS, ubiquitin-proteasome system.

There are several possible cellular and molecular mechanisms that underlie the benefits of the multimodal intervention on protein metabolism. On the one hand, the concurrent exercise and leucine supplementation may accelerate MPS directly through PI3K/Akt/mTOR dependent pathway. Moreover, Akt inhibits the functional expression of FOXO and its downstream signaling cascade. On the other hand, the multimodal treatment may also decrease MPB by attenuating circulating inflammatory cytokines. Then the FOXO dependent signaling cascade, including atrogene transcription and ubiquitin-proteasome system activation, will both be inhibited (D'Antona and Nisoli, 2010). Consequently, protein balance in skeletal muscle will tend to be positive, and atrophy will be lessened (Figure 2).

## Conclusions and future directions

The influence of sarcopenia on physical and metabolic functions is a significant concern in the public health and scientific community. The underlying etiopathogenesis of sarcopenia appears multifaceted and not yet fully defined, but ultimately involves the gradual decrease of MPS and increase of MPB. Core goals of sarcopenia treatment should include ensuring sufficient protein intake, maintaining skeletal muscle mass and function, and reducing low-grade inflammation where present. From a functional point of view, sarcopenia is often accompanied by decrements in muscle strength that increase the incidence of fractures and falls that often result in prolonged hospitalizations and a further loss of independence. To address these risks, resistance exercises focusing on increasing not only muscle mass but also muscle strength are highly indicated. On the other hand, metabolic disorders that occur partly as a result of hypokensia are frequently associated with the aging process and contribute to the occurrence of sarcopenia via multiple mechanisms. Therefore, reducing the incidences of these associated metabolic disorders may be necessary to attenuate the progression of muscle loss associated with aging and the development of sarcopenia. To address these concerns, low and/or moderate intensity aerobic exercise seems to be effective to restore insulin sensitivity, reduce inflammation, and decrease adiposity. To make these exercise interventions multimodal, nutritional support is needed. It is well-accepted that individuals performing aerobic or resistance exercises require higher protein intakes than sedentary individuals, and older adults may benefited from increasing leucine consumption. Future research in humans should better establish how the dose of leucine (vs. other amino acids) in relation to daily protein intake, energy intake and different exercise regimens (resistance exercise alone, aerobic exercise alone or concurrent training) affects the quality of muscle during the middle years of life (35–55 years of age) as well as during the aging process (>65 years of age). Additional studies may then investigate to what extent and by which mechanisms protein synthesis and degradation might be directly involved in sarcopenia pathogenesis and the multimodal treatment responses.

## Author contributions

Substantial contributions to the conception or design of the work: ZX, YZ, HS, and NZ. Drafting the work or revising it critically for important intellectual content: ZX, JC, YZ, YY, KA, QS, FL. Final approval of the version to be published: ZX, JC, NZ. All authors agree to be accountable for the content of the work.

### Conflict of interest statement

The authors declare that the research was conducted in the absence of any commercial or financial relationships that could be construed as a potential conflict of interest.
